# Elevated circulating Hsp70 levels are correlative for malignancies in different mammalian species

**DOI:** 10.1007/s12192-022-01311-y

**Published:** 2022-11-18

**Authors:** Lukas Salvermoser, Krzysztof Flisikowski, Susann Dressel-Böhm, Katarzyna J. Nytko, Carla Rohrer Bley, Angelika Schnieke, Ann-Kathrin Samt, Dennis Thölke, Philipp Lennartz, Melissa Schwab, Fei Wang, Ali Bashiri Dezfouli, Gabriele Multhoff

**Affiliations:** 1 Radiation Immuno-Oncology Group, Center for Translational Cancer Research Technische, Universität München (TranslaTUM), Technische Universität München (TUM), Klinikum Rechts Der IsarEinsteinstr 25, 81675 Munich, Germany; 2 Department of Radiation Oncology, Klinikum Rechts Der Isar, Technische Universität München (TUM), Ismaningerstr 22, 81675 Munich, Germany; 3 Department of Radiology, University Hospital, LMU Munich, Marchioninistr 15, 81377 Munich, Germany; 4 Livestock Biotechnology, School of Live Sciences, Technische Universität München (TUM), Liesel-Beckmannstr 1, 85354 Freising, Germany; 5 Vetsuisse Faculty, Division of Radiation Oncology, University of Zurich, Winterthurerstr 258C, CH-8057 Zurich, Switzerland

**Keywords:** CompHsp70 ELISA, Extracellular Hsp70, Tumor biomarker, Tumor animal model, Osteosarcoma, Colorectal polyps

## Abstract

Circulating Hsp70 levels were determined in feline and porcine cohorts using two different ELISA systems. These comparative animal models of larger organisms often reflect diseases, and especially malignant tumors, better than conventional rodent models. It is therefore essential to investigate the biology and utility of tumor biomarkers in animals such as cats and pigs. In this study, levels of free Hsp70 in the blood of cats with spontaneously occurring tumors were detected using a commercial Hsp70 ELISA (R&D Systems). Sub-analysis of different tumor groups revealed that animals with tumors of epithelial origin presented with significantly elevated circulating Hsp70 concentrations. In addition to free Hsp70 levels measured with the R&D Systems Hsp70 ELISA, levels of exosomal Hsp70 were determined using the compHsp70 ELISA in pigs. Both ELISA systems detected significantly elevated Hsp70 levels (R&D Systems: median 24.9 ng/mL; compHsp70: median 44.2 ng/mL) in the blood of a cohort of *APC*^*1311/*+^ pigs diagnosed with high-grade adenoma polyps, and the R&D Systems Hsp70 ELISA detected also elevated Hsp70 levels in animals with low-grade polyps. In contrast, in fl*TP53*^*R167H*^ pigs, suffering from malignant osteosarcoma, the compHsp70 ELISA (median 674.32 ng/mL), but not the R&D Systems Hsp70 ELISA (median 4.78 ng/mL), determined significantly elevated Hsp70 concentrations, indicating that in tumor-bearing animals, the dominant form of Hsp70 is of exosomal origin. Our data suggest that both ELISA systems are suitable for detecting free circulating Hsp70 levels in pigs with high-grade adenoma, but only the compHsp70 ELISA can measure elevated, tumor-derived exosomal Hsp70 levels in tumor-bearing animals.

## Introduction

As molecular chaperones, heat shock proteins (HSPs) play crucial roles in the protein homeostasis of cells by preventing protein aggregation, supporting correct folding of nascent polypeptides, coordinating the assembly and disassembly of macromolecular structures, transporting proteins across membranes, regulating cell cycle and apoptosis, and facilitating antigen processing (Vabulas et al. 2010; Selvarajah et al. 2013; Craig 2018; De Maio and Hightower 2021). The 72-kDa major stress-inducible member of the heat shock protein 70 family Hsp70 (HSPA1A) belongs in the group of best studied stress proteins (Mayer and Bukau 2005; Madden et al. 2008; Radons 2016; Sekhar et al. 2016; Rosenzweig et al. 2019). While the synthesis of most cellular proteins is downregulated during and following nonlethal stress, the synthesis of HSPs and especially that of Hsp70 is highly upregulated. Apart from elevated temperatures as a classical inducer, a large variety of different stress stimuli including nutrient deficiency, hypoxia followed by reoxygenation, amino acid analogs, heavy metals, UV light, ionizing irradiation, and cytostatic and anti-inflammatory drugs induce the stress protein synthesis. Under physiological conditions, cell growth, differentiation, and cell maturation induce the heat shock/stress response. Interestingly, cellular Hsp70 levels in tumor cells are generally higher than those in normal cells, even in the absence of stress (Hantschel et al. 2000; Ciocca and Calderwood 2005; Rohde et al. 2005; Akerfelt et al. 2010; Pockley and Henderson 2018) to protect cells from apoptosis and mediate therapy resistance. HSPs inhabit nearly all subcellular compartments and our group was among the first who demonstrated a tumor cell–specific surface expression of Hsp70 on a large variety of malignant cell types of human and murine origin (Multhoff et al. 1995; Hantschel et al. 2000; Stangl et al. 2011a, b). The cell membrane localization of Hsp70 on human tumor cells is enabled by a tumor-specific sphingoglycolipid composition mediating membrane Hsp70 anchorage (Gehrmann et al. 2008). Viable, membrane Hsp70–positive tumor cells have the capacity to actively release the major stress-inducible Hsp70 (HSPA1A) in extracellular lipid microvesicles (Cordonnier et al. 2017) with physicochemical properties of exosomes (Gastpar et al. 2005). However, most commercially available Hsp70 ELISA systems detect only free Hsp70 (predominantly in aqueous solutions) which most likely originates from dying cells, whereas the compHsp70 ELISA, which employs two monoclonal antibodies (cmHsp70.1, cmHsp70.2), enables the quantification of both free Hsp70 and exosomal Hsp70 in liquid biopsies (Werner et al. 2021).

Since the amino acid sequence of Hsp70 is highly conserved across different mammalian species (Hartl 1996; Daugaard et al. 2007; Shalgi et al. 2014; Radons 2016), the cmHsp70.1 and cmHsp70.2 monoclonal antibodies used in the compHsp70 ELISA are able to detect Hsp70 not only in the blood of human cancer patients, but also in tumor-bearing rodents and canines (Breuninger et al. 2014; Bayer et al. 2014; Salvermoser et al. 2019; Rothammer et al. 2019; Werner et al. 2021). Moreover, a reduction of viable tumor mass upon radiation therapy has been shown to be associated with decreased levels of circulating Hsp70 in mice (Bayer et al. 2014). Therefore, we speculate that circulating Hsp70 levels might serve as a useful tumor biomarker to monitor therapeutic response in a broad range of experimental and clinical settings.

To the best of our knowledge, Hsp70 concentrations have yet not been assessed in the blood of tumor-bearing pet animals such as cats. This is surprising since spontaneous occurring tumors in comparative medicine reflect the human disease much better than many rodent tumor models for the following reasons: pet animals live in the same (non-germfree) environment as humans, receive high-quality healthcare, and their tumors show a high genetic heterogeneity and develop spontaneously at a greater age, like most human tumors (Paulin et al. 2018; Rossa and D’Silva 2019; Nascimento and Ferreira 2021; Hassan et al. 2017; Vozenin et al. 2019).

Due to the high physiological and anatomical similarities such as organ size and a comparatively long lifespan, pigs provide increasingly popular and informative tumor models (Flisikowska et al. 2016; Boas et al. 2020; Nurili et al. 2021; Rossa and D’Silva 2019). Different disease stages of human colorectal cancer from low-grade to high-grade polyps as well as human osteosarcomas can be mimicked by genetic engineering. Porcine osteosarcoma models helped to elucidate novel pathways to study this tumor entity on a molecular level (Sieren et al. 2014; Saalfrank et al. 2016; Tanihara et al. 2018; Jarvis et al. 2021).

However, the use of comparative animal models in cancer research is often hampered by the lack of tumor-specific reagents such as antibodies with specificities for different animal species. Most antibodies directed against tumor biomarkers are certified only for frequently used laboratory animals such as mice and rats. Although a few studies have investigated SDF-1 (Marques et al. 2017), PD-1, and PD-L1 (Nascimento et al. 2020) as potential tumor biomarkers in feline mammary carcinoma, no published studies exist for relevant tumor-specific biomarkers in pigs. Considering that blood samples can easily and repeatedly be obtained by minimally invasive methods and in a time- and cost-effective manner, the measurement of blood-derived tumor biomarkers by ELISA provides an attractive tool in clinical medicine. We demonstrated that the Hsp70 sequence recognized by the Hsp70-specific antibodies cmHsp70.1 (aa 454–461, N-L-L-G-R-F-E-L) (Stangl et al. 2011a, b) and cmHsp70.2 (aa 614–623, A-G-G-P-G-G-F-G) (Werner et al. 2021) of the compHsp70 ELISA are highly conserved in humans and many other animal species including mice, rats (Stangl et al. 2011a, b; Breuninger et al. 2014), dogs (Salvermoser et al. 2019), horses, cows, cats, and pigs (Werner et al. 2021). Therefore, it was reasonable to assume that this ELISA has the capacity to measure free and exosomal Hsp70 in the blood of different animal species.

The aim of the present study was to evaluate the predictive value of circulating free (as detected by the R&D Systems Hsp70 ELISA) and exosomal Hsp70 (as detected by the compHsp70 ELISA/Hsp70-exoELISA) in different animal species as a potential biomarker for pre-cancerous lesions and malignant tumors.

## Materials and methods

### Animals

To assess circulating Hsp70 in felines, EDTA blood of 79 cats was collected by venous blood draw; 41 animals served as a healthy control cohort (Table 1) and 38 cats were suffering from different malignant tumors. According to their cytological characteristics (Raskin and Meyer 2016; Withrow et al. 2012; Villiers et al. 2016), 8 animals had mesenchymal tumors, 21 epithelial tumors, and 9 round cell tumors (Table 2).

**Table 1:** Characteristics of the feline control cohort (*n* = 41)

No	Breed	Age (years)	Weight (kg)	Sex
1	European shorthair	0.5	3.4	Male
2	European shorthair	0.5	3.2	Male
3	European shorthair	15.8	5.1	Male
4	European shorthair	11.2	3.1	Male
5	European shorthair	12.2	7.8	Male
6	European shorthair	13.9	5.0	Female
7	European shorthair	11.2	4.6	Male
8	European shorthair	1.2	–	Male
9	European shorthair	5.3	3.3	Male
10	Sphinx cat	10.0	2.5	Female
11	European shorthair	4.7	5.6	Female
12	European shorthair	11.1	6.6	Male
13	European shorthair	9.7	3.7	Male
14	European shorthair	6.7	3.8	Female
15	European shorthair	5.3	3.0	Female
16	Bengal cat	5.1	3.5	Female
17	European shorthair	9.1	6.8	Male
18	European shorthair	2.6	5.6	Female
19	European shorthair	2.6	5.8	Male
20	European shorthair	13.9	3.5	Female
21	European shorthair	3.1	5.3	Male
22	European shorthair	11.5	5.9	Male
23	European shorthair	11.5	6.4	Male
24	European shorthair	3.7	5.0	Male
25	European shorthair	8.2	6.5	Female
26	European shorthair	9.5	5.2	Female
27	European shorthair	5.8	7.6	Male
28	European shorthair	8.9	6.1	Female
29	European shorthair	18.2	2.4	Female
30	European shorthair	2.5	4.0	Male
31	Maine coon	1.4	5.7	Female
32	Maine coon	3.7	6.9	Male
33	Maine coon	13.3	4.2	Female
34	Maine coon	1.0	5.8	Male
35	Maine coon	5.7	5.3	Female
36	Maine coon	7.1	6.9	Male
37	Maine coon	8.7	7.2	Male
38	European shorthair	8.2	4.8	Male
39	Norwegian forest cat	13.2	2.8	Female
40	European shorthair	6.2	4.6	Male
41	European shorthair	7.2	5.9	Female

**Table 2:** Characteristics of the feline tumor cohort (*n* = 38)

No	Breed	Age (years)	Weight (kg)	Sex	Diagnosis	Tumor origin	Tumor location	Extension
1	European shorthair	9.6	4.3	Male	Hemangiosarcoma	Mesenchymal	Ball of foot	Local
2	European shorthair	16.2	4.6	Male	Hemangiosarcoma	Mesenchymal	Nasal cavity	Local
3	European shorthair	7.9	4.3	Female	Fibrosarcoma	Mesenchymal	Thoracic wall	Local
4	European shorthair	12.5	8.3	Male	Fibrosarcoma	Mesenchymal	Shoulder, neck, thoracic wall	Local
5	European shorthair	12.4	5.5	Male	Fibrosarcoma	Mesenchymal	Shoulder	Local
6	European shorthair	15.7	3.1	Female	Fibrosarcoma	Mesenchymal	Interscapular	Systemic
7	European shorthair	10.9	4.2	Female	Fibrosarcoma	Mesenchymal	Lip, mandible, lung	Systemic
8	European shorthair	15.6	4.9	Male	Osteosarcoma (extraskeletal)	Mesenchymal	Abdominal wall	Systemic
9	European shorthair	15.1	2.7	Female	Adenocarcinoma	Epithelial	Nasal cavity	Local
10	British shorthair	4.4	5.7	Female	Adenocarcinoma	Epithelial	Tail	Local
11	European shorthair	12.6	7.4	Male	Adenocarcinoma	Epithelial	Nasal cavity	Local
12	European shorthair	11.1	4.5	Female	Adenocarcinoma	Epithelial	Nasal cavity	Local
13	Siberian cat	13.7	5.2	Female	Adenocarcinoma	Epithelial	Mamma	Local
14	European shorthair	13.5	4.0	Male	Adenocarcinoma	Epithelial	Nasal cavity, piriform lobe	Systemic
15	British longhair	17.8	2.7	Female	Salivary gland tumor	Epithelial	Parotid gland	Local
16	Siamese cat	7.9	3.8	Male	Squamous cell ca	Epithelial	Oral mucosa	Local
17	European shorthair	12.3	7.8	Male	Squamous cell ca	Epithelial	Oral mucosa	Local
18	European shorthair	16.7	2.9	Female	Squamous cell ca	Epithelial	Nose	Local
19	Siamese cat	14.2	5.0	Female	Squamous cell ca	Epithelial	Oral mucosa	Local
20	Persian cat	15.7	5.2	Male	Squamous cell ca	Epithelial	Oral mucosa	Local
21	European shorthair	8.4	4.8	Male	Squamous cell ca	Epithelial	Nose	Local
22	Maine coon/ European shorthair	12.9	5.8	Male	Squamous cell ca	Epithelial	Cheek	Local
23	European shorthair	9.9	4.6	Female	Squamous cell ca	Epithelial	Sublingual	Local
24	European shorthair	12.0	8.1	Male	Squamous cell ca	Epithelial	Mandible	Local
25	European shorthair	10.5	4.4	Female	Squamous cell ca	Epithelial	Nasal cavity	Local
26	European shorthair	9.4	4.3	Male	Squamous cell ca (in situ)	Epithelial	Ear, nose, eye	Local
27	European shorthair	10.5	4.3	Male	Squamous cell ca (in situ)	Epithelial	Nose, ear	Local
28	European shorthair	7.0	5.0	Female	Squamous cell ca (in situ)	Epithelial	Nose	Local
29	Siamese cat	12.8	7.0	Male	Thymoma	Epithelial	Intrathoracic	Local
30	European shorthair	9.0	5.1	Male	Mast cell tumor	Round cell	Forehead, eye	Systemic
31	Birman cat	0.7	2.6	Female	Lymphoma	Round cell	Mediastinum	Local
32	European shorthair	4.8	4.0	Female	Lymphoma	Round cell	Nasal cavity	Local
33	European shorthair	14.9	5.2	Female	Lymphoma	Round cell	Scapula	Local
34	Maine coon	12.2	6.5	Male	Lymphoma	Round cell	Nasal cavity	Local
35	Siamese cat	11.7	4.7	Female	Lymphoma	Round cell	Nasal cavity	Local
36	European shorthair	12.9	3.6	Female	Lymphoma	Round cell	Nasal cavity	Local
37	European shorthair	14.0	4.7	Male	Lymphoma	Round cell	Oral cavity, nose, toe	Systemic
38	Birman cat	11.3	3.9	Male	Lymphoma	Round cell	Pancreas, jejunum, mediastinum	Systemic

The porcine cohort consisted of 30 healthy control animals, and 48 *APC*^*1311/*+^ animals that had a diagnosis of colorectal polyps classified into low-grade (*n* = 22) and high-grade adenoma polyps (*n* = 26) (Flisikowski et al. 2022). The *APC*^*1311/*+^ mutant pigs contain a G to A substitution leading to a premature stop codon at position 1311 of porcine tumor suppressor adenomatous polyposis coli (APC). The mutation APC^1311^ in pigs is orthologous to the hotspot APC^1309^ in humans which causes severe familial adenomatous polyposis. *APC*^*1311/*+^ pigs recapitulate the major hallmarks of the human disease including the varying severity of polyposis, even between siblings (Flisikowski et al. 2022). As a tumor model, fl*TP53*^*R167H*^ pigs (*n* = 24) diagnosed with osteosarcoma were included in the study (Niu et al. 2021a, b). The fl*TP53*^*R167H*^ pigs contain a latent R167H mutation which is homologous to the hotspot R175H oncogenic mutation in the human p53. All homozygous and the majority of heterozygous pigs with the non-recombined fl*TP53*^*R167H*^ allele develop osteosarcoma by the age of 18 months. Due to several competing studies, only a limited amount of blood was available and therefore not all animals of the osteosarcoma cohort could be analyzed using both ELISA tests.

The study was carried out in strict accordance to the recommendations and an approved protocol of the Animal Ethics Councils of the Canton of Zurich, Switzerland (permit numbers ZH172/13 and ZH065/16) for cats and the Government of Upper Bavaria, Germany (permit number 55.2–2532.Vet_02-18–33) for pigs. All animal experiments were performed under the supervision of an experienced veterinarian and according to the Swiss and German Animal Welfare Act and European Union Normative for Care and Use of Experimental Animals.

### R&D Systems Hsp70 ELISA

Free circulating Hsp70 levels in plasma samples from the feline and porcine cohort were measured using the R&D Systems commercial DuoSet IC Human/Mouse/Rat total Hsp70 ELISA (DYC1663E; R&D Systems, Minneapolis, MN) according to the manufacturer’s instructions. The R&D ELISA determines only the inducible Hsp70 (HSP70A1A) and does not cross-react with the following recombinant human HSP70 proteins such as GRP78, HSPA2, HSPA6, and HSPA8.

### CompHsp70 ELISA

Free and exosomal Hsp70 levels in plasma samples from the porcine cohort were measured using the compHsp70 ELISA, as described by Werner et al. (2021). Briefly, a 96-well MaxiSorp Nunc-Immuno plate (Thermo, Rochester, NY) was coated overnight by incubating with 1 μg/mL cmHsp70.2 (multimmune GmbH, Munich, Germany) in 100 µL sodium carbonate buffer (0.1 M sodium carbonate, 0.1 M sodium hydrogen carbonate, pH 9.6; Sigma-Aldrich). Next, the plate was washed with phosphate-buffered saline (PBS; Life Technologies) supplemented with 0.05% v/v Tween-20 (Calbiochem, Merck, Darmstadt, Germany). Blocking was performed by incubation with Blocking Solution (300 µL; Candor Bioscience GmbH, Wangen im Allgäu, Germany) for 30 min at room temperature. After another washing step, samples were diluted at 1:15 for canine and porcine and 1:5 for human and canine in StabilZyme Select Stabilizer (Diarect GmbH, Freiburg im Breisgau, Germany) and 100 µL added to the wells. Plates incubated for 30 min at room temperature. An eight-point concentration standard curve of Hsp70 protein (0–100 ng/mL) diluted in StabilZyme Select Stabilizer (Diarect GmbH) was included in each assay. Following another washing step, wells were incubated with 200 ng/mL in 100 µL of the biotinylated cmHsp70.1 monoclonal antibody (mAb) (multimmune GmbH) diluted in HRP-Protector (Candor Bioscience GmbH) for 1 h at room temperature. After a final washing step, plates were incubated for 30 min at room temperature with 57 ng/mL in 100 µL horseradish peroxidase (HRP)–conjugated streptavidin (Senova GmbH, Weimar, Germany) diluted in HRP-Protector (Candor Bioscience GmbH). After washing, plates were incubated with substrate reagent (BioFX TMB Super Sensitive One Component HRP Microwell Substrate; Surmodics, Inc., Eden Prairie, MN) for 15 min at room temperature. The colorimetric reaction was stopped by adding 2 N H_2_SO_4_ and absorbance read at 450 nm, corrected by absorbance at 570 nm, in a microplate reader (VICTOR X4 Multilabel Plate Reader; PerkinElmer, Waltham, MA). The values given include the dilution factors of the samples. The Hsp70-exo ELISA (EIA-6201; DRG Instruments GmbH, Marburg, Germany) which is based on the monoclonal antibodies cmHsp70.1 and cmHsp70.2 (multimmune GmbH) delivers comparable results to those of the compHsp70 ELISA.

### Dynamic light scattering

Exosomes derived from serum/plasma were characterized with respect to size and homogeneity by dynamic light scattering (DLS) technique using a Zetasizer Nano SZ instrument (Malvern Panalytical, Malvern, UK) at 25 °C with a refractive index of 1.38.

### Exosome depletion

Plasma of a porcine and of human donors with different circulating Hsp70 levels were used for exosome depletion. Exosomes were depleted from the blood samples by ultracentrifugation (47,000 rpm, 2 h, 4 °C). Approval for the blood draw of human donors was obtained by the Institutional Ethical Review Board. Written informed consent was obtained from the tumor patient and the healthy volunteers included into the study before blood was taken by venous puncture.

### Western blot analysis of exosomes and cell lysates

Tumor cells (LS174T, human colon carcinoma cells; K9STS, canine sarcoma cell line, kindly provided by Dr. Carla Rohrer-Bley) were cultured in RPMI-1640 medium supplemented with exosome-depleted FCS (10%) up to 70% confluency. Cells (LS174T, peripheral blood lymphocytes of pigs) and purified exosomes derived from FCS were diluted in radioimmunoprecipitation assay (RIPA) buffer containing 50 mM Tris–HCl (pH 8.0), 150 mM NaCl, 1 mM EDTA, 1% v/v Triton X, 0.1% w/v sodium dodecyl sulfate (SDS), 0.5% w/v sodium deoxycholate, and protease inhibitor (Roche, Basel, Switzerland). Exosomes were ultrasonicated (UP200S; Hielscher Ultrasonics GmbH, Teltow, Germany) for 2 min at room temperature. After running on a SDS-PAGE, proteins were transferred on nitrocellulose membranes and detected by immunoblotting with the following antibodies directed against Hsp70 (cmHsp70.1, IgG1; multimmune GmbH), β-actin (A2228; Sigma-Aldrich, St. Louis, MO, USA), and HRP-conjugated rabbit anti-mouse immunoglobulins (P0260; DakoAgilent, Santa Clara, CA, USA). Immune complexes were visualized by a Pierce ECL Western kit (Thermo Fisher Scientific, Waltham, MA, USA) and imaged digitally (ChemiDoc Touch Imaging System; BioRad, Hercules, CA, USA).

### Immunophenotyping of exosomes

Exosomes derived from FCS and the supernatant of a canine sarcoma cell line (K9STS), diluted in PEB (PBS + 5 mM EDTA + 0.5% bovine serum albumin (BSA)) buffer were immunophenotyped by flow cytometry using FITC-conjugated Hsp70 (cmHsp70.1; multimmune GmbH), PE-conjugated CD9 (Clone SN4 C3-3A2; Miltenyi Biotec, Bergisch Gladbach, Germany), and APC-conjugated CD63 (Clone H5C6; Miltenyi Biotec) monoclonal antibodies (mAbs) for 1 h at 4 °C. The incubation period of the exosomes with the fluorescence-labeled antibodies was 15 min in the dark. After another two washing steps, stained exosomes were analyzed on a MACS Quant flow cytometer (Miltenyi Biotech).

### Statistics

Hsp70 values were measured at least three times with the two ELISA systems described above. Mean values were calculated for each individual animal and considered for statistical analysis.

As the distribution of Hsp70 data was skewed, median values and quartiles are presented as median with the first and third quartile. For comparison of independent samples, the Mann–Whitney *U* test was performed. The Mann–Whitney *U* test was used to compare paired data. All statistical tests were performed two-sided and a significance level of α = 5% was applied.

Pearson’s correlation coefficient (*r*) and *R*^2^ values were calculated with a poor correlation assumed for *r* values not exceeding + 0.2/ − 0.2, a fair correlation for *r* values not exceeding + 0.5/ − 0.5, a moderate correlation for *r* values not exceeding + 0.7/ − 0.7, a very strong correlation for *r* values not exceeding + 0.9/ − 0.9, and a perfect correlation for an *r* value of + 1 or − 1 (Chan 2003; Akoglu 2018; Bishara and Hittner 2012). SPSS Statistics version 27.0 (IBM, Armonk, NY) was used to perform statistical analysis. GraphPad Prism version 9.0 (GraphPad, San Diego, CA) was used for graphing.

## Results

### Free Hsp70 levels are increased in cats with epithelial tumors

**Fig. 1:**
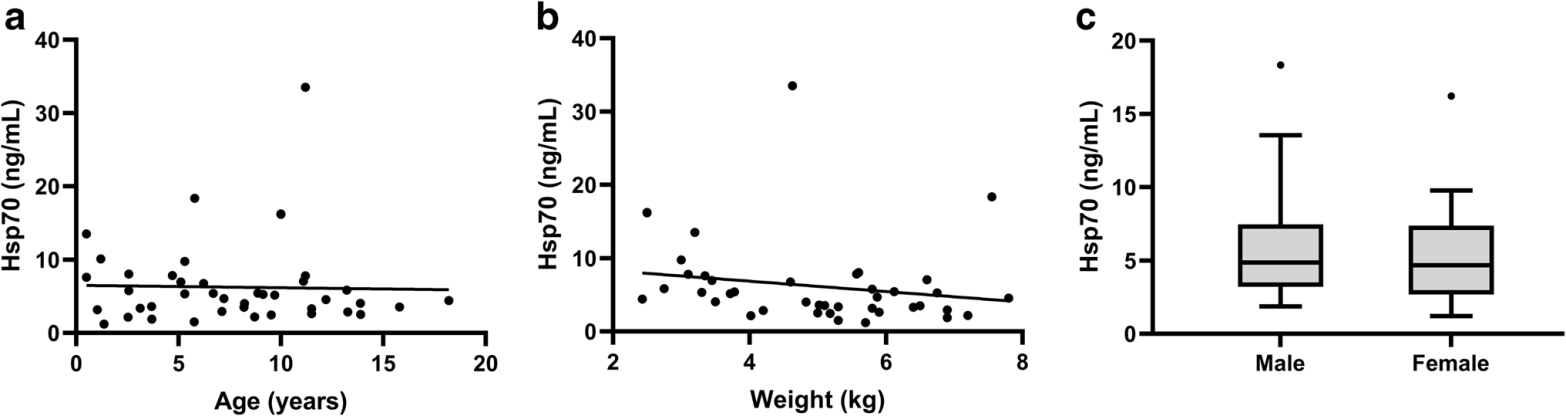
Free Hsp70 concentrations, as determined using the R&D Systems Hsp70 ELISA in the blood of tumor-free cats (*n* = 41) were compared to age (**a**), weight (**b**), and gender (24 male vs. 17 female) (**c**). Lines inside the box plots show the median value, upper and lower boundaries indicate the 25th and 75th percentiles, and whiskers indicate highest and lowest value within 1.5 IQR, respectively. Not all outliers shown. *p < 0.05, Mann–Whitney *U* test

To exclude a potential influence of age and body weight on circulating Hsp70 levels in domestic cats, free Hsp70 was measured in a tumor-free control cohort. The biological characteristics of the animals such as breed, age, weight, and gender that were admitted to the veterinary clinic for routine examinations such as vaccination, hip X-ray, castration, or sterilization are summarized in Table 1. Pearson’s correlation coefficients (*r*) of Hsp70 concentration versus age (*n* = 41) and weight (*n* = 40) were − 0.001 and − 0.183, respectively, indicating a poor correlation (Fig. 1a, b). The corresponding *R*^2^ value was 0.000003 and 0.0337 for age and weight, respectively. These data show that the two variables do not correlate with free Hsp70 concentrations in the tumor-free control cohort. No correlation was also found with respect to the gender of the animals. Differences in Hsp70 values in the blood of male (*n* = 24) and female (*n* = 17) cats did not reach statistical significance (*p* = 0.853) (Fig. 1c).

**Fig. 2:**
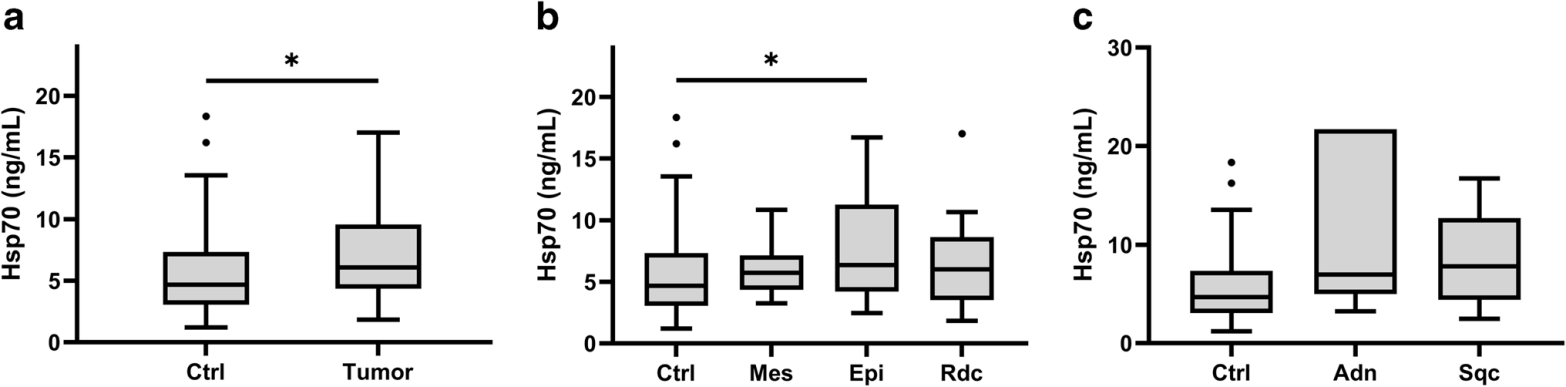
Free Hsp70 concentrations in the blood of tumor-free (Ctrl: *n* = 41) and tumor-bearing (Tumor: *n* = 38) cats, as determined using the R&D Systems Hsp70 ELISA (**a**). Free Hsp70 concentrations in the blood of tumor-free cats (*n* = 41) and cats with tumors of epithelial (Epi: *n* = 21; *p* = 0.043) and mesenchymal (Mes: *n* = 8; *p* = 0.34) origin and round cell tumors (Rdc: *n* = 9; *p* = 0.46) (**b**). Free Hsp70 concentrations in the blood of tumor-free cats (*n* = 41) and cats with adenocarcinomas (Adn: *n* = 6; *p* = 0.109) and squamous cell carcinomas (Sqc: *n* = 10; *p* = 0.055) (**c**). Lines inside the box plots show the median value, upper and lower boundaries indicate the 25th and 75th percentiles, and whiskers indicate highest and lowest value within 1.5 IQR, respectively. Not all outliers shown. *p < 0.05, Mann–Whitney *U* test

Table 2 summarizes the breed, age, weight, gender, diagnosis, histological characteristics, location, and extension of each individual tumor-bearing cat. The tumors were classified into mesenchymal (*n* = 8), epithelial (*n* = 21), and round cell tumors (*n* = 9). Tumors of mesenchymal origin include different types of sarcoma and chondroma, epithelial tumors include squamous cell and adenocarcinomas, and round cell tumors include lymphoma and mast cell tumors. Animals with carcinoma in situ were excluded from the study. Previous studies have shown that human patients with different tumor types (Breuninger et al. 2014; Werner et al. 2021) and domestic dogs suffering from round cell tumors (Salvermoser et al. 2019) exhibit significantly elevated Hsp70 concentrations in the blood when compared to a healthy control cohort. Therefore, Hsp70 concentrations of all tumor-bearing cats (*n* = 38) were compared to that of a tumor-free control cohort (*n* = 41). In line with our previous findings and as illustrated in Fig. 2a, Hsp70 levels were significantly higher in cats with tumors of different entities compared to tumor-free control animals (*p* = 0.045). Although all three types of tumors revealed elevated median free Hsp70 levels, significantly different Hsp70 values were only observed in cats with epithelial tumors (*n* = 21; *p* = 0.043) (Fig. 2b). This result might be due to the relatively low numbers of cats with mesenchymal (*n* = 8) and round cell tumors (*n* = 9). Furthermore, elevated median Hsp70 levels were found in cats with adenocarcinoma (Adn; *n* = 6; median: 6.96 ng/mL) and squamous cell carcinoma (Sqc; *n* = 10; median: 7.80 ng/mL) when in situ carcinomas were excluded (Fig. 2c). However, also in this cohort of animals the differences failed to reach statistical significance due to the relatively small sample size (Adn: *p* = 0.109; Sqc: *p* = 0.055).

### An inter-species comparison of different healthy mammalians shows highest free circulating Hsp70 concentrations in cats

**Fig. 3:**
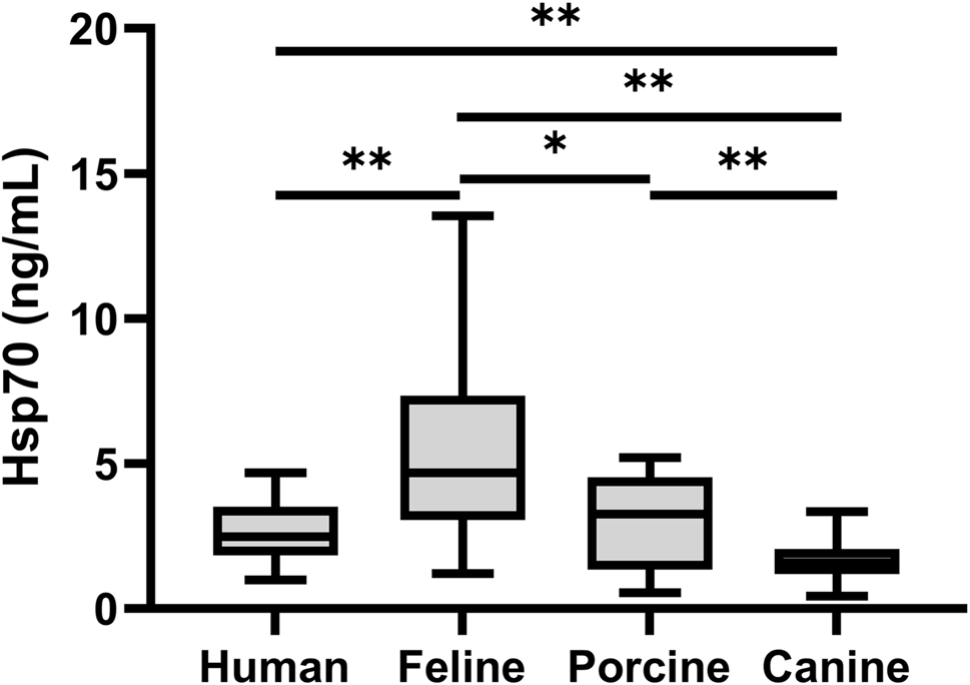
Free Hsp70 concentrations in the blood of healthy human volunteers (*n* = 108), tumor-free cats (*n* = 41), pigs (*n* = 30), and dogs (*n* = 38), as determined using the R&D Systems Hsp70 ELISA. Lines inside the box plots show the median value, upper and lower boundaries indicate the 25th and 75th percentiles, and whiskers indicate highest and lowest value within 1.5 IQR, respectively. Outliers not shown. **p < 0.01; *p < 0.05, Mann–Whitney *U* test

Figure 3 illustrates an inter-species comparison of free Hsp70 concentrations in human volunteers and different mammalian species, as determined using the R&D Systems Hsp70 ELISA. The median Hsp70 concentration in human blood was 2.49 ng/mL (Werner et al. 2021). In tumor-free cats, pigs, and dogs, the median Hsp70 concentrations were 4.69, 3.27, and 1.57 ng/mL, respectively. Consequently, the basal Hsp70 concentration in cats was significantly higher than that of the other three species (feline vs. human: *p* = 0.001; feline vs. porcine: *p* = 0.027; feline vs. canine: *p* = 0.001). With a median concentration of 3.27 ng/mL, Hsp70 levels in the blood of pigs are significantly lower than those in cats but significantly higher than those in dogs (*p* = 0.002) and comparable to those in humans (*p* = 0.319). Salvermoser et al. (2019) have previously reported that healthy dogs present with a median Hsp70 concentration of 1.57 ng/mL and therefore these values are significantly lower than those in humans (*p* = 0.001).

### Free and exosomal Hsp70 levels are increased in pigs with high-grade colorectal adenoma polyps

The R&D Systems Hsp70 ELISA only detects free Hsp70, whereas the compHsp70 ELISA is able to measure both free Hsp70 derived from dying cells and vesicular Hsp70 which is actively released by viable tumor cells in exosomes (Werner et al. 2021). Both Hsp70 ELISA systems specifically detect the inducible Hsp70 (HSPA1A) and do not cross-react with other chaperones or other members of the HSP70 family. According to the manufacturer’s instructions, the R&D ELISA recognizes only the inducible Hsp70 (HSPA1A) and does not cross-react with the following recombinant human HSP70 proteins such as GRP78, HSPA2, HSPA6, and HSPA8. The compHsp70 ELISA consists of two Hsp70-specific monoclonal antibodies cmHsp70.1 (amino acid, aa 457–463, N-L-L-G-R-F-E-L) and cmHsp70.2 (aa 614–623, A-G-G-P-G-P-G-G-F-G) which are highly specific for the inducible form of Hsp70 (HSPA1A). Both antibodies do not cross-react with the constitutive form Hsc70 (HSPA8) or other chaperones as determined by Western blot analysis of tumor cell lysates and by dot blot analysis using Hsp27, Hsp60, and Hsp90 as recombinant proteins (Werner et al. 2021). A comparison of the epitope recognized by the cmHsp70.1 antibody, used as a detection antibody in the compHsp70 ELISA, reveals no amino acid exchange in the Hsp70 epitope sequence of humans, mice, rats, dogs, bovines, horses, and pigs (Werner et al. 2021). The epitope of cmHsp70.2, used as a coating antibody, exhibits 1 amino acid exchange (position 619 from P to A) in the human versus dog, bovine, and horse Hsp70 epitope sequence, and 2 amino acid exchanges between the human and rodent epitope sequence (position 616 from G to A and position 619 from P to A). No amino acid exchange exists in the Hsp70 epitope sequence of cmHsp70.2 between humans and pigs. With respect to cats, only the sequence of HSPA2 but not that of HSPA1 (Hsp70) is publicly available.

**Fig. 4:**
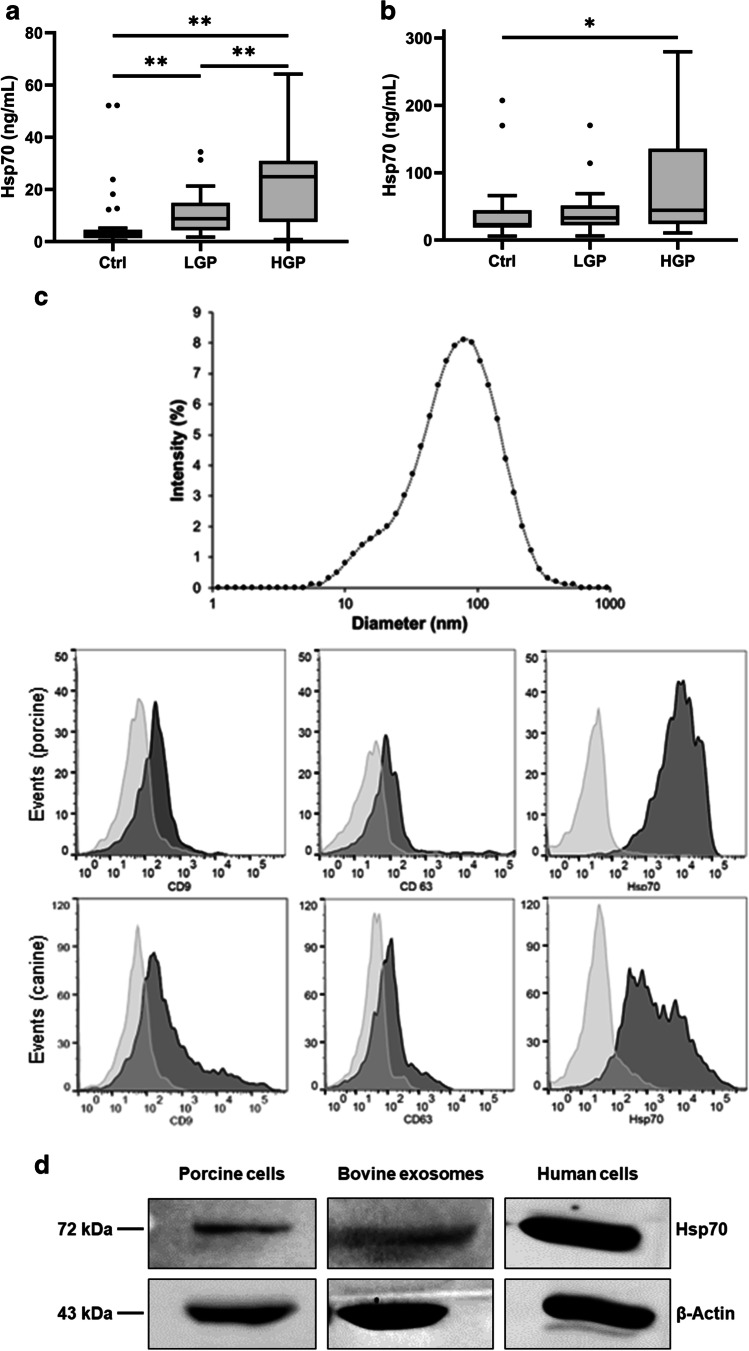
Free and exosomal Hsp70 concentrations in the blood of *APC*^*1311/*+^ pigs diagnosed with low-grade adenoma polyps (LGP: *n* = 22), high-grade adenoma polyps (HGP: *n* = 26), and a healthy control cohort (Ctrl: *n* = 30). Free Hsp70 concentrations determined using the R&D Systems Hsp70 ELISA were significantly higher in LGP and HGP animals compared to the control group (**a**). Exosomal Hsp70 concentrations measured using the compHsp70 ELISA were significantly higher in HGP animals compared to the control group (**b**). Lines inside the box plots show the median value, upper and lower boundaries indicate the 25th and 75th percentiles, and whiskers indicate highest and lowest value within 1.5 IQR, respectively. Not all outliers are shown. **p < 0.01, *p < 0.05, Mann–Whitney *U* test. Size distribution (upper panel) and typical exosomal cell surface markers in extracellular vesicles derived from porcine cells (middle panel) and a canine sarcoma cell line (K9STS) (lower panel), as determined by dynamic light scattering (DLS) (**c**). The surface of extracellular vesicles was stained with antibodies directed against Hsp70 (cmHsp70.1-FITC) and typical exosomal markers such as CD9 (anti-CD9-PE) and CD63 (anti-CD63-APC). Representative histograms of the respective markers are shown in gray, the isotype-matched controls are shown in white. Western blot analysis of exosomes derived from fetal calf serum (FCS) and cell lysates of pig lymphocytes and a human tumor cell line (LS174T) (**d**). The blot was stained with the cmHsp70.1 monoclonal antibody, β-actin served as a loading control; the molecular weights are indicated

To investigate the value of Hsp70 as a prognostic tumor biomarker, free and exosomal Hsp70 concentrations were measured in healthy control pigs and genetically modified *APC*^*1311/*+^ animals with low-grade (*n* = 22) and high-grade (*n* = 26) colorectal adenoma polyps. Animals with low- and high-grade adenoma polyps exhibited significantly elevated free Hsp70 concentrations when compared to a healthy control cohort (*p* = 0.0001). As illustrated in Fig. 4a, free Hsp70 concentrations of pigs with low- (*p* = 0.004) and high-grade (*p* = 0.0001) colorectal polyps differed significantly from that of the control group.

Measurements of free and exosomal Hsp70 levels using the compHsp70 ELISA revealed more than fivefold higher Hsp70 concentrations in healthy pigs compared to those measured with the R&D Systems Hsp70 ELISA (median compHsp70 ELISA: 22.9 ng/mL; median R&D Hsp70 ELISA: 3.3 ng/mL). Animals with high-grade polyps, but not with low-grade polyps, exhibited significantly elevated Hsp70 levels when measured with the compHsp70 ELISA (*p* = 0.023) (Fig. 4b). For high-grade polyps, the Hsp70 levels in the blood measured with the compHsp70 ELISA were almost twice as high compared to those measured with the R&D Systems Hsp70 ELISA (median compHsp70 ELISA: 44.2 ng/mL; median R&D Hsp70 ELISA: 24.9 ng/mL). This finding might indicate that approximately half of the circulating Hsp70 in the blood origins from exosomal Hsp70. To prove that the compHsp70 ELISA detects both exosomal and free Hsp70, Hsp70 levels were measured in untreated plasma and exosome-depleted plasma derived from the blood of a tumor patient, human volunteers with different basal Hsp70 values, and a porcine donor. As shown in Table 3, Hsp70 levels dropped drastically after exosome depletion in all samples. Since it is nearly impossible to resuspend the exosomal pellet fraction in ELISA buffer properly, ELISA data of the exosomal fraction were not provided. In a previous study, biophysical characteristics of exosomes were determined by the presence of cytosolic chaperones such as Hsc70, the nucleotide exchange factor Bag4 (Johnson and Gestwicki 2022) and the lysosomal marker Rab4 and the absence of ER residing chaperones such as Grp94 or calnexin in the lumen of the exosomes, a size of the microvesicles in the range of 50 to 100 nm, and a peak in the acetyl-choline esterase activity at a density of 1.17 g/mL (Gastpar et al. 2005). Since these antibodies which are used for human exosomal markers show no cross-reactivity with the antigens of other mammalian species, these markers could not be determined on exosomes derived from other mammalian species apart from humans. However, the exosomal nature of extracellular vesicles isolated from porcine cells and the supernatant of a canine sarcoma cell line (K9STS) was shown by the typical size distribution in the range of 50 to 100 nm, as measured by dynamic light scattering (Fig. 4c, upper panel), and by the presence of Hsp70 and other typical exosomal markers such as CD9 and CD63 on the surface of the extracellular microvesicles, as determined by flow cytometry (porcine cells: Fig. 4c, middle panel; canine tumor cells: Fig. 4c, lower panel). To demonstrate that the cmHsp70.1 monoclonal antibody used as a detection antibody in the compHsp70 ELISA recognizes exosomal Hsp70, exosomes isolated from bovine calf serum by ultracentrifugation were subjected to an SDS-PAGE. Western blot analysis using the cmHsp70.1 antibody reveals a 72-kDa band in the exosomal fraction of bovines. Cell lysates of porcine peripheral blood lymphocytes and a human tumor cell line (LS174T) were used as a molecular weight control (Fig. 4d).

**Table 3:** Hsp70 values measured with the compHsp70 ELISA in the plasma of a human tumor patient, healthy human volunteers with different basal Hsp70 values, and porcine plasma before (untreated) and after exosome depletion

Donor information	Untreated	Exosome depleted
Human plasma (tumor)	120.9	14.8
Human plasma (healthy)	52.29	21.99
Human plasma (healthy	9.34	5.2
Human plasma (healthy)	3.52	2.53
Porcine plasma	32.3	5.5

### High exosomal but not free Hsp70 levels are predictive for genetically induced osteosarcoma in pigs

**Fig. 5:**
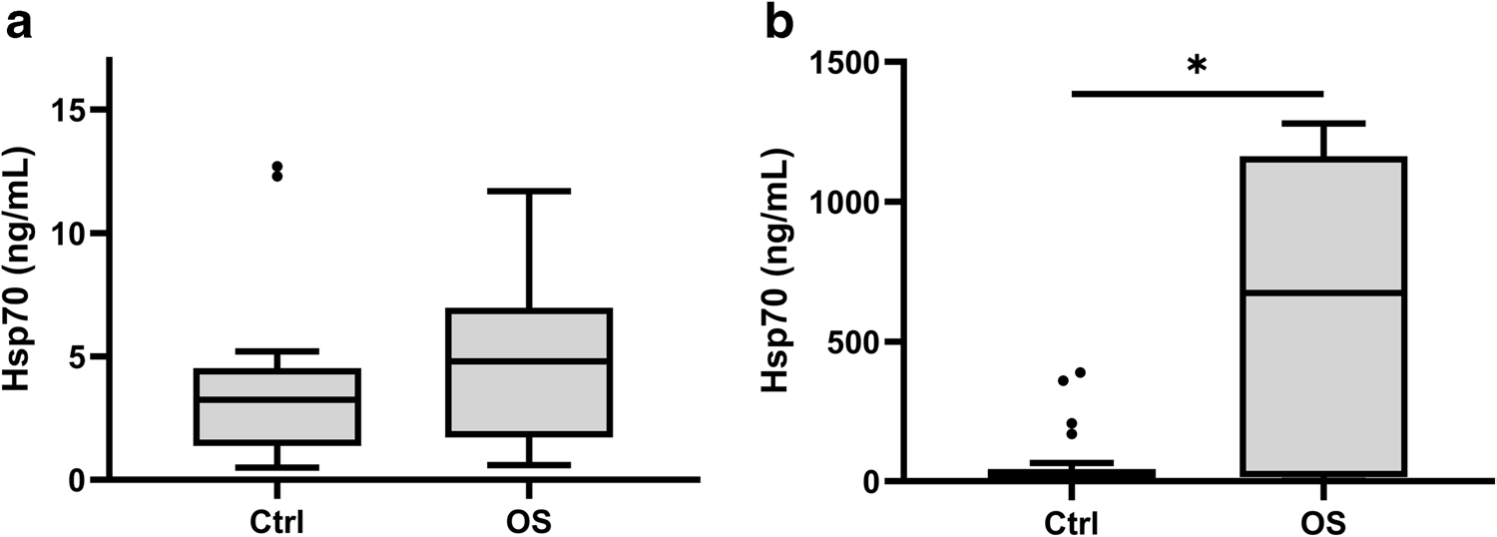
Free and exosomal Hsp70 concentrations of fl*TP53*^*R167H*^ pigs diagnosed with osteosarcoma. Free Hsp70 concentrations measured with the R&D Systems Hsp70 ELISA did not significantly differ from those in healthy controls (Ctrl: *n* = 30) and animals with osteosarcoma (OS: *n* = 16) (**a**). Exosomal Hsp70 concentrations measured using the compHsp70 ELISA in the blood of healthy controls (Ctrl: *n* = 30) and animals with osteosarcoma (OS: *n* = 24) differed significantly (**b**). Lines inside the box plots show the median value, upper and lower boundaries indicate the 25th and 75th percentiles, and whiskers indicate highest and lowest value within 1.5 IQR, respectively. Not all outliers shown. *p < 0.05, Mann–Whitney *U* test

Genetically modified *TP53*^*R167H*^ pigs develop osteosarcoma that mimics the clinical situation in human patients with osteosarcoma. Free Hsp70 concentrations, as determined using the R&D Systems Hsp70 ELISA (*n* = 16) in *TP53*^*R167H*^ pigs diagnosed with osteosarcoma, were compared to those in healthy control animals. As shown in Fig. 5a, the differences in free Hsp70 in both groups were very low (median values ranged between 3 and 5 ng/mL) and differences between control animals and osteosarcoma-bearing animals did not reach statistical significance (*p* = 0.166). Moreover, levels of free Hsp70 in tumor-bearing pigs were lower than those in pigs with low- and high-grade polyps, indicating that free Hsp70 in the circulation does not reflect the viable tumor mass.

In contrast, the compHsp70 ELISA, which detects free and exosomal Hsp70 in the blood, shows significantly elevated Hsp70 concentrations in osteosarcoma-bearing animals (*p* = 0.022; *n* = 24) compared to control animals, as outlined in Fig. 5b. As the values measured with the compHsp70 ELISA were more than 100-fold higher compared to those measured with the R&D Systems Hsp70 ELISA (median compHsp70 ELISA: 674.32 ng/mL; median R&D Hsp70 ELISA: 4.78 ng/mL), we assume that in malignant neoplasia such as osteosarcoma, predominantly exosomal Hsp70 derived from viable tumor cells contribute to the elevated Hsp70 levels. Animals suffering from high-grade premalignant lesions had tenfold lower exosomal Hsp70 levels than tumor-bearing animals.

## Discussion

Although many certified reagents such as antibodies are available for mice and rats, the clinical relevance of data derived from rodent tumor models for human diseases is limited. Due to their longer lifespan, a not completely germ-free living environment, and a life-long medical care, cats and pigs qualify as comparative animal models that reflect the clinical situation of cancer in humans much better than rodents. All tumor-bearing cats recruited into our study suffered from spontaneously occurring malignant tumors at an older age, a condition that depicts the human situation very closely. Moreover, the suitability of genetically modified pig models for studying premalignant lesions in colorectal cancer and malignant osteosarcoma has been demonstrated in several previous studies (Niu et al. 2021a,b; Ehrenfeld et al. 2022; Yim et al. 2021; Flisikowski et al. 2022; Rogalla et al. 2019; Troya et al. 2021).

To the best of our knowledge, very few studies have reported on the presence and utility of biomarkers in liquid biopsies of felines and porcines. The role of extracellular Hsp70 as a universal tumor biomarker has become of increasing interest over the past decades.

An elevated Hsp70 expression on tumor cells has been shown to serve as a biomarker for tumor aggressiveness, therapeutic response, and overall survival (Pockley and Henderson 2018; Gunther et al. 2015; Chanteloup et al. 2020). Extracellular vesicles derived from tumor cells with high intracellular and membrane bound Hsp70 levels subsequently contain more Hsp70 in their lumen and on the exosomal surface (Gastpar et al. 2005). Circulating Hsp70 levels have shown potential as a valuable biomarker in various human cancer entities including colorectal, lung, and mammary carcinoma (Breuninger et al. 2014; Werner et al. 2021) and can predict its progressive capacity (Jagadish et al. 2016; Krawczyk et al. 2020; De Freitas et al. 2022). In breast and non-small cell lung carcinomas, the Hsp70 positivity of the blood was associated with higher tumor stages and an increased risk for developing metastases (Rothammer et al. 2019; Gunther et al. 2015). Circulating Hsp70 values provide a more valuable predictive marker to distinguish patients with metastatic and non-metastatic disease than the enumeration of circulating tumor cells (Chanteloup et al. 2020) and serve as a predictive marker for prognosis (Hsu et al. 2022). Extracellular Hsp70 is enhancing tumor growth and migration by promoting multiple pro-oncogenic pathways including the PI3K/Akt and phosphorylated STAT3 pathways (Lee et al. 2006; Diao et al. 2015; Park et al. 2017). Moreover, extracellular Hsp70 levels have been found to affect tumor associated immune responses (Linder and Pogge von Strandmann 2021; Pockley and Henderson 2018) and exosomal Hsp70 acts as a regulator of tumor-associated immune cells which determines immunosurveillance and evasion (Multhoff et al. 2001; Lee et al. 2006; Specht et al. 2015; Barreca et al. 2017; Taha et al. 2019).

In animals, malignant canine round cell tumors (Salvermoser et al. 2019), rodent squamous cell carcinoma of the head and neck, and ductal adenocarcinoma of the pancreas (Stangl et al. 2011a, b; Bayer et al. 2014) are associated with elevated circulating Hsp70 levels. Due to the high sequence homology in different mammalian species, it was assumed that Hsp70 might also have utility as a biomarker in cats and pigs.

In the feline cohort, free Hsp70 was measured in the circulation with the R&D Systems Hsp70 ELISA. Age and weight of the animals could be excluded as a confounding factor that affects Hsp70 levels since Hsp70 concentrations did not show any correlation with age or weight in the tumor-free control cohort. A comparison of Hsp70 levels in control animals and tumor-bearing animals revealed significantly elevated Hsp70 concentrations in tumor-bearing cats and pigs. This finding accounts for the entirety of the tumor cohort, as well as for cats with epithelial tumors. Although the sub-groups of animals with adenocarcinomas and squamous cell carcinomas showed trends toward higher Hsp70 concentrations, the differences did not reach statistical significance. Most likely, the small cohort size accounts for this observation. Although all other main tumor groups (mesenchymal origin, round cell tumors) showed elevated median Hsp70 values, the data failed to show statistical significance. It could be that the tumor size was too small and/or the amount of released Hsp70 was below the detection limit of the ELISA. Moreover, exosomal Hsp70 may play a more important role than free Hsp70 in the detection of tumors by biomarkers in liquid biopsies.

It has been shown previously that extracellular Hsp70 levels in the blood are linked to tumor size and volume in mouse models (Bayer et al. 2014) and in human patients with NSCLC (Gunther et al. 2015). Since the tumor size was not assessed in spontaneous feline tumor patients, a correlation between tumor size/volume and circulating Hsp70 was not possible in this study. However, these data are essential for a better understanding of the role of Hsp70 levels in the blood as a predictive/prognostic tumor biomarker.

An inter-species comparison of free Hsp70 concentrations in tumor-free animals revealed significantly higher basal levels in felines. The reason for the higher basal Hsp70 concentrations in cats compared to that of human volunteers and canines (Breuninger et al. 2014; Werner et al. 2021; Salvermoser et al. 2019) remains to be elucidated. It is well accepted that the metabolism in cats differs from that of other mammals. Cats have a higher physiological body temperature and metabolize many drugs faster than other species (Court 2013; Brodeur et al. 2017; Al-Dabbagh and Smith 1984). In line with these observations, one might speculate that a higher metabolic demand (Strage et al. 2021; Geddes et al. 2016) and an elevated body temperature (Calderwood et al. 2006; De Maio 2014; Radons 2016; Kiraly et al. 2020) might contribute to the higher Hsp70 concentrations in the circulation of cats.

In the porcine cohort, Hsp70 was measured in the blood using two different ELISA systems, the compHsp70 ELISA which detects free and exosomal Hsp70 and the R&D Systems Hsp70 ELISA which only detects free Hsp70. Overall, free Hsp70 levels remained at very low concentrations in pigs with malignant osteosarcoma, and no significant differences in free Hsp70 levels in tumor-bearing and healthy animals could be determined. In contrast, the Hsp70 concentrations measured with the compHsp70 ELISA were 100-fold higher than those measured with the R&D Systems Hsp70 ELISA and tumor-bearing animals exhibited significantly elevated exosomal Hsp70 levels compared to control animals. This finding could be explained by the ability of the compHsp70 ELISA to detect both free and exosomal Hsp70 in the circulation, the latter originating from viable tumor cells.

Both ELISA systems were able to detect elevated Hsp70 levels in high-grade colorectal polyps. However, the compHsp70 ELISA showed higher levels than the R&D Systems Hsp70 ELISA, and significant differences were only observed between healthy pigs and pigs with high-grade polyps. The R&D Systems Hsp70 ELISA showed significant differences in levels of free Hsp70 levels in control versus low-grade and versus high-grade polyp groups. However, it is essential to note that free Hsp70 levels were higher in animals with polyps compared to animals with malignant tumors. In contrast, exosomal Hsp70 levels measured with the compHsp70 ELISA were significantly higher in tumor-bearing animals than in animals with low- and high-grade polyps and healthy controls. This finding might indicate that exosomal Hsp70, which is actively released by living tumor cells, better reflects the presence of malignant tumors than free Hsp70 derived from dying cells and is therefore more relevant as a tumor biomarker.

The exosomal nature of extracellular vesicles derived from the blood of animals and a canine tumor cell line was shown by the size distribution in the range of 50–100 nm, as measured by dynamic light scattering, and by the presence of typical exosomal surface markers such as the tetraspanines CD9, CD63, and Hsp70. Other chaperones and exosomal markers could not be determined due to the lack of antibodies that show cross-reactivity to these antigens in different mammalian species.

In conclusion, the present study was able to show that free Hsp70 in the circulation of cats and especially exosomal Hsp70 in pigs have potential as promising tumor biomarkers. Although other studies have already demonstrated the potential of Hsp70 in the blood of humans and rodents as a biomarker for a large variety of tumors, the present study extended these observations to feline and porcine species. Similar to our previous studies, the compHsp70 ELISA demonstrated its ability to detect not only free but also exosomal Hsp70 in the circulation of tumor-bearing animals. However, further investigations with larger animal cohorts which also include the analysis of the tumor size before and after therapy are necessary to determine the potential of circulating (exosomal) Hsp70 levels as a tumor biomarker in different animal species and to judge the utility of this biomarker for monitoring therapeutic response.
