# Correction to: Elevated circulating Hsp70 levels are correlative for malignancies in different mammalian species

**DOI:** 10.1007/s12192-022-01315-8

**Published:** 2022-11-29

**Authors:** Lukas Salvermoser, Krzysztof Flisikowski, Susann Dressel-Böhm, Katarzyna J. Nytko, Carla Rohrer Bley, Angelika Schnieke, Ann-Kathrin Samt, Dennis Thölke, Philipp Lennartz, Melissa Schwab, Fei Wang, Ali Bashiri Dezfouli, Gabriele Multhoff

**Affiliations:** 1 Radiation Immuno-Oncology Group, Center for Translational Cancer Research Technische, Universität München (TranslaTUM), Technische Universität München (TUM), Klinikum Rechts Der IsarEinsteinstr 25, 81675 Munich, Germany; 2 Department of Radiation Oncology, Klinikum Rechts Der Isar, Technische Universität München (TUM), Ismaningerstr 22, 81675 Munich, Germany; 3 Department of Radiology, University Hospital, LMU Munich, Marchioninistr 15, 81377 Munich, Germany; 4 Livestock Biotechnology, School of Live Sciences, Technische Universität München (TUM), Liesel-Beckmannstr 1, 85354 Freising, Germany; 5 Vetsuisse Faculty, Division of Radiation Oncology, University of Zurich, Winterthurerstr 258C, CH-8057 Zurich, Switzerland

**Correction to: Cell Stress and Chaperones**

**https://doi.org/10.1007/s12192-022-01311-y**

In the original version of this article, the first author's given and family names were transposed and should have read Lukas Salvermoser.

The original article has been corrected.

